# Enrichment and characterization of a bacterial culture that can degrade 4-aminopyridine

**DOI:** 10.1186/1471-2180-13-62

**Published:** 2013-03-21

**Authors:** Shinji Takenaka, Ryosuke Nomura, Ayumi Minegishi, Ken-ichi Yoshida

**Affiliations:** 1 Department of Applied Biological Chemistry, Graduate School of Agricultural Science, Kobe University, 1-1 Rokkodai, Nada-ku, Kobe 657-8501, Japan

**Keywords:** 4-aminopyridine, 4-amino-3-hydroxypyridine, 3,4-dihydroxypyridine, *Hyphomicrobium*, *Pseudomonas nitroreducens*, *Elizabethkingia*

## Abstract

**Background:**

The agrichemical 4-aminopyridine is used as a bird repellent in crop fields and has an epileptogenic action in a variety of animals, including man and mouse. 4-Aminopyridine is biodegraded in the environment through an unknown mechanism.

**Results:**

A 4-aminopyridine-degrading enrichment culture utilized 4-aminopyridine as a carbon, nitrogen, and energy source, generating 4-amino-3-hydroxypyridine, 3,4-dihydroxypyridine, and formate as intermediates. 4-Amino-3-hydroxypyridine could not be further metabolized and probably accumulated as a dead-end product in the culture. Biodegradability tests and partial sequence analysis of the enrichment culture indicated that 4-aminopyridine was mainly degraded via 3,4-dihydroxypyridine and that the metabolite is probably cleaved by 3-hydroxy-4-pyridone dioxygenase. Seven culturable predominant bacterial strains (strains 4AP-A to 4AP-G) were isolated on nutrient agar plates. Changes in the bacterial populations of 4-aminopyridine, 3,4-dihydroxypyridine, or formate/ammonium chloride enrichment cultures were monitored by denaturing gradient gel electrophoresis (DGGE) profiling of PCR-amplified 16S rRNA gene fragments. Sequence analysis of the 16S rRNA gene fragments derived from predominant DGGE bands indicated that *Pseudomonas nitroreducens* 4AP-A and *Enterobacter* sp. 4AP-G were predominant in the three tested enrichment cultures and that the unculturable strains *Hyphomicrobium* sp. 4AP-Y and *Elizabethkingia* sp. 4AP-Z were predominant in 4-aminopyridine and formate/ammonium chloride enrichment cultures and in the 3,4-dihydroxypyridine enrichment culture, respectively. Among the culturable strains, strain 4AP-A could utilize 3,4-dihydroxypyridine as a growth substrate. Although we could not isolate strain 4AP-Y on several media, PCR-DGGE analysis and microscopy indicated that the unique bi-polar filamentous bacterial cells gradually became more dominant with increasing 4-aminopyridine concentration in the medium.

**Conclusions:**

*Hyphomicrobium* sp. 4AP-Y, *P. nitroreducens* 4AP-A, and *Elizabethkingia* sp. 4AP-Z probably play important roles in 4-aminopyridine degradation in crop fields. In the enrichment culture, 3,4-dihydroxypyridine and its metabolites including formate might be shared as growth substrates and maintain the enrichment culture, including these indispensable strains.

## Background

Pyridine and its derivatives are mainly produced on an industrial scale from coal tar. These compounds are major industrial raw materials and intermediates used for organic solvents and the production of agrichemicals, medicines, and active surfactants [1]. Pyridines are soluble in polar and nonpolar solvents, and most are toxic [2].

Pyridine and its derivatives are also environmental pollutants, and their biodegradation has been studied in detail [3]. The biodegradability of pyridine derivatives follows the order pyridinecarboxylic acids > pyridine = monohydroxypyridines > methylpyridines > aminopyridines = chloropyridines [4]. Generally, pyridines are degraded via pyridine-ring reduction and fission steps [5] or via pyridine-ring hydroxylation and fission steps [6-8]. *Nocardia* sp. strain Z1 directly cleaves the pyridine ring between N and position C-2 and further metabolizes the product via glutaric dialdehyde, and *Bacillus* sp. strain 4 cleaves the ring between positions C-2 and C-3 and the product it further via succinate semialdehyde [9]. *Gordonia nitida* strain LE31 metabolizes 3-methyl- or 3-ethyl-pyridine without a hydroxylation step [5]. *Rhodococcus opacus* (VKM Ac-1333D) and *Arthrobacter crystallopoietes* (VKM Ac-1334D) hydroxylate the pyridine ring [8]. In *Agrobacterium* sp. strain NCIB 10413, 4-hydroxypyridine is metabolized by a hydroxylase and an *N*-heterocyclic ring-cleavage dioxygenase [6,7]. Thus, the biodegradation of pyridines by single bacterial species has been studied, but little is known about the biodegradation of pyridines by microbial communities [10], which could include unculturable bacteria.

**Figure 1:**
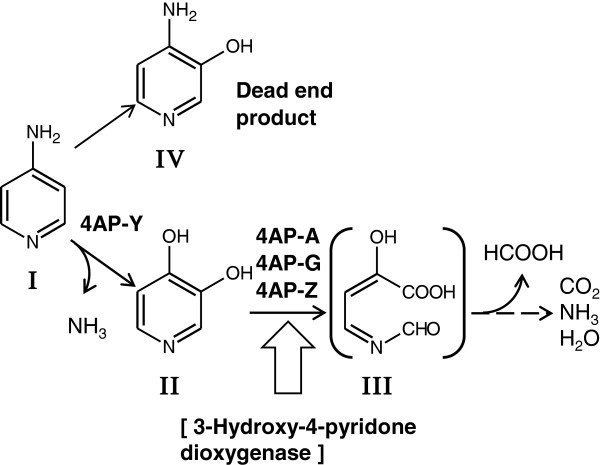
**Proposed pathway of 4-aminopyridine degradation by the enrichment culture.** I, 4-aminopyridine; II, 3,4-dihydroxypyridine; III, 3-(*N*-formyl)-formiminopyruvate; and IV, 4-amino-3-hydroxypyridine. The ring-cleavage product 3-(*N*-formyl)-formiminopyruvate from 3,4-dihydroxypyridine was hypothesized from the metabolic pathway of 3,4-dihydroxypyridine in *Agrobacterium* sp. NCIB 10413 [6,7]. The strains of the enrichment culture probably involved in the steps are indicated.

Aminopyridines are persistent chemical [4] and are a class of potentially genotoxic impurities in pharmaceutical products [11]. 4-Aminopyridine (Figure 1, compound I) has been marketed for agricultural use as Avitrol and used for repelling and killing bird pests [12]. The compound is a potassium-channel blocker [13] and has epileptogenic action in a variety of animals, including man and mouse [14,15]. However, the metabolic fate of 4-aminopyridine in an ecosystem [16] and its biodegradation by an isolated a bacterium or bacterial community has not been studied in detail. It is broken down slowly by soil microorganisms in 2 months [16]. Here we report the enrichment and adaptation of a 4-aminopyridine-degrading enrichment culture and the characterization of the bacterial populations under different culture conditions.

## Methods

### Organisms and growth conditions

Enrichments of 4-aminopyridine-degrading bacteria were set up with 0.2 g normal farm soils such as rice field soil and corn field soils from the Hyogo Prefecture, Japan in 7 ml basal medium containing 2.13 mM (0.02% wt/vol) 4-aminopyridine as described previously [17]. Briefly, solutions A (sodium-potassium phosphate solution), B (metal-salt solution containing 1 ml of a soil extract), and C (4-aminopyridine solution) were prepared separately. The soil extract used in solution B was prepared by adding 15 g of a normal rice field soil to 200 ml of deionized water and mixing for 30 min, followed by filtration through Whatman No. 2 filter paper (Maidstone, UK) and autoclaving.

Ten 4-aminopyridine-degrading enrichment cultures, KM20-14A to KM20-14J, were incubated at 30°C with shaking at 140 rpm. Every 4 days, 500 μl of the enrichment culture was used to inoculate 7 ml fresh medium, to maintain 4-aminopyridine degradation ability. We selected one enrichment culture derived from a normal rice field soil, No. KM 20-14E for further study and examined its utilization of the identified metabolites (4-amino-3-hydroxypyridine and 3,4-hydroxypyridine) by the enrichment culture (No. KM20-14E) was examined. The tested substrate was added to the basal medium instead of 4-aminopyridine.

### Isolation and identification of culturable and unculturable strains from the 4-aminopyridine-degrading enrichment culture

Samples taken from the 4-aminopyridine-degrading enrichment culture were serially diluted 10^6^- to 10^8^-fold with 0.8% (wt/vol) NaCl solution and spread onto nutrient agar plates (1.0 g polypeptone, 1.0 g meat extract, 0.5 g NaCl, and 1.5 g agar per 100 ml), 0.1% (wt/vol) 4-aminopyridine agar plates, and 0.1% (wt/vol) 3,4-dihydroxypyridine agar plates. The plates were incubated at 30°C for 4 to 7 days, and colonies were picked up for 16S rRNA gene analysis. We designated seven dominant bacterial strains isolated from the nutrient agar plate as dominant bacterial strains 4AP-A to 4AP-G. The 16S rRNA gene V3 regions derived from these strains were used as a PCR-DGGE analysis makers as described below.

The isolates were characterized by physiological and biochemical parameters, such as gram reaction, flagella type, catalase activity, oxidase activity, OF test, fluorescent pigment production, and hydrolysis of gelatin, starch, and urea, following classical methods and by 16S rRNA gene analysis [18] (see Additional file 1: Tables S1 and S2). Minor or unculturable strains were classified only by 16S rRNA gene analysis. 16S rRNA genes were amplified using the universal primers pA and pH’ [18] (Table 1), and their nucleotide sequences (approximately 1,500 bp) were determined and compared to sequences in the DDBJ/EMBL/GenBank database.

**Table 1:** Oligonucleotide primers used in this study

**Primer**	**Sequence (5' to 3')**	**Reference**
pA	AGAGTTTGATCCTGGCTCAG	[7]
	(8–28)	
pH’	AAGGAGGTGATCCAGCCGCA	[7]
	(1542–1522)	
PRBA338GCf	CGCCCGCCGCGCGCGGCGGGCGGGGCGGGGGCACGGGGGGACTCCTACGGGAGGCAGCAG	This study
PRBA338f	TACGGGAGGCAGCAG	[26]
PRUN518r	ATTACCGCGGCTGCTGG	[26]
PRSTY1 ^*a*^	ACGATAATGACGGTACCCGG	This study
PRSTY2 ^*a*^	TTAGCCGGGACTTATTCTCC	This study
PRSTZ1 ^*b*^	TACTTACGTGTAAGTAGCTGAAGG	This study
PRSTZ2 ^*b*^	CCTTCAGCTACTTACACGTAAGTA	This study
PydAf ^*c*^	GAYGAYCAYTTYGARAAYCA	This study
PydAr ^*c*^	CATICCRCADATCCAYTC	This study

^*a*^ Used for amplification of the full-length 16S rRNA gene from strain 4AP-Y.

^*b*^ Used for amplification of the full-length 16S rRNA gene from strain 4AP-Z.

^*c*^ PydAf and PydAr were designed based on the conserved regions of 3-hydroxy-4-pyridone dioxygenase (3,4-dihydroxypyridine 2,3-dioxygenase), DDHFENH and EWICGM, respectively. R is A or G; Y is C or T; D is A, G, or T; and I is inosine.

### Isolation, and identification of metabolites from 4-aminopyridine

The enrichment culture was cultivated in basal medium containing 2.13 mM 4-aminopyridine at 30°C with shaking, and the culture was diluted 10^6^ to 10^8^-fold with 0.8% (wt/vol) NaCl solution. The diluted culture (500 μl) was used to inoculate fresh basal medium containing 4-aminopyridine, and the subculture was incubated at 30°C. The culture was centrifuged at 20,000 × *g* for 10 min, and the supernatant was dried using a rotary evaporator. The dried residues were dissolved in *n*-butanol and then dried again. The accumulated products in the dried residue were incubated with *N,O*-bis(trimethylsilyl)trifluoroacetamide at 100°C for 1.5 h. The trimethylsilylated products were analyzed by GC-MS as described below.

### Measurement and identification of 4-aminopyridine and its metabolites

Concentrations of pyridines, including 4-aminopyridine and 4-amino-3-hydroxypyridine (Figure 1, compound IV), were measured using a Hitachi L-6200 HPLC system (Tokyo, Japan) equipped with a Cosmosil 5C18 PAQ column (4.6 × 150 mm; Nacalai Tesque, Kyoto). The eluent was 20 mM potassium phosphate buffer (pH 2.5) containing 5 mM pentanesulfonate; the flow rate was 1.0 ml/min. 4-Aminopyridine and 4-amino-3-hydroxypyridine were detected at 254 nm and had retention times of 5.4 and 7.6 min, respectively. The metabolites from 4-aminopyridine (4-amino-3-hydroxypyridine and 3,4-dihydroxypyridine; Figure 1) were identified and quantified using a GCMS-QP2010 Ultra (Shimadzu, Kyoto, Japan). A fused silica capillary column (InertCap 1MS; 0.25 mm × 30 m; GL Science) was used. Helium gas was the carrier at a linear velocity of 35 cm/s. The column temperature was programed from 50°C (held for 1 min) to 280°C at a rate of 5°C/min and then held at 280°C for 20 min. The peaks derived from the trimethylsilylated derivatives of 4-aminopyridine, 4-amino-3-hydroxypyridine, and 3,4-dihydroxypyridine appeared at 18.2, 24.5, and 20.9 min, respectively. The organic acids in the culture supernatant were derivatized by pentafluorobenzyl bromide according to a previously reported method [19] and analyzed by GC-MS as described above. The peaks derived from the pentafluorobenzyl formate appeared at 8.5 min.

### PCR-DGGE analysis

(1) DNA extraction and PCR

Aliquots (1.5, 1.0, and 0.5 ml) of the enrichment culture were sampled at the early-, mid-, and late-exponential growth phases, respectively, and centrifuged. DNA in the cell pellets was extracted using Qiagen DNeasy Blood & Tissue Kit according to the manufacturer’s instructions (Nihon eido, Tokyo, Japan). The 16S rRNA genes were amplified from 0.5 μl DNA by PCR (50 μl reactions) using a Taq polymerase kit (TaKaRa BIO INC., Shiga, Japan) and the forward primer PRBA338GCf, which contains a GC clamp, and the reverse primer PRUN518r, which targets the V3 region of the 16S rRNA gene (Table 1); the primers were prepared as reported previously [20]. The following PCR protocol was used: initial denaturation at 95°C for 2 min; 35 cycles of denaturation at 95°C for 60 s, annealing at 60°C for 30 s, extension at 72°C for 30 s; and final extension at 72°C for 5 min. The 16S rRNA genes of isolated strains were amplified by PCR of DNA isolated from colonies.

(2) DGGE

Approximately 100 to 200 ng of each PCR product was analyzed by electrophoresis on 1.0-mm-thick polyacrylamide [8% (wt/vol) acrylamide-bis-acrylamide (779:21)] DGGE gels following the manufacturer’s protocol (Nihon Eido Co. Ltd., Tokyo, Japan). The denaturing gradient was from 27.5 to 42.5% [100% corresponded to 7.08 M urea and 40% (wt/vol) formamide]. Gels were subjected to a constant voltage of 50 V for 4 h at 60°C. After electrophoresis, the gels were stained for 20 min in ethidium bromide solution. DNA was visualized under UV light, digitally captured, and analyzed using a Gel Imaging System (Nippon Genetics Co. Ltd., Tokyo, Japan).

(3) Cloning of PCR product and sequencing

Prominent DNA bands from the DGGE gels were extracted and used as PCR templates with the forward primer PRBA338f without a GC clamp and the reverse primer PRUN518r. The nucleotide sequences obtained were compared with those of the 16S rRNA genes of the strains isolated. To analyze the full-length 16S rRNA gene sequences, specific primers were designed based on the partial sequences of the isolate that became more dominant in the culture during continuous growth in basal medium containing 4-aminopyridine (Table 1).

### PCR amplification of part of the 3-hydroxy-4-pyridone dioxygenase gene

The enrichment culture grown in 4-aminopyrdine-containing medium was harvested in the mid-exponential growth phase by centrifugation. Mixed genomic DNA in the cell pellets was extracted using Qiagen DNeasy Blood & Tissue Kit (Hilden, Germany) according to the manufacturer’s instructions and was used as a template for PCR. To amplify part of the 3-hydroxy-4-pyridone dioxygenase (3,4-dihydroxypyridine 2,3-dioxygenase) gene, *pydA*, the primers PydAf and PydAr were designed based on the conserved region of previously reported dioxygenases from *Rhizobium* sp. TAL1145 (DDBJ/EMBL/GenBank accession no. AY729020), *Hyphomicrobium* sp. MC1 (YP_004673996), *Bordetella bronchiseptica* RB50 (NP_890665), and *Bordetella parapertussis* 12822 (NP_885852) (Table 1). The following PCR protocol was used: initial denaturation at 95°C for 2 min; 35 cycles of denaturation at 95°C for 60 s, annealing at 45°C for 30 s, extension at 72°C for 30 s; and final extension at 72°C for 5 min. Harvesting of cells, preparation of mixed genomic DNA, and amplification were carried out in triplicate.

### Analytical methods

The optical density (OD_660_) of the cultures was measured using a Hitachi U-2800 spectrophotometer. The ^1^H-NMR spectra of the isolated metabolites and the prepared standard compounds were measured with a Joel JNM-AL300 spectrometer (300 MHz, Joel Ltd., Tokyo, Japan). Released ammonia in the culture fluid was measured using the indophenol blue method [21]. Total protein in the culture was measured using the modified Lowry method, to confirm the utilization of 4-aminopyridine as a carbon, nitrogen, and energy source by the enrichment culture [22].

### Nucleotide sequence accession numbers

The nucleotide sequences of the 16S rRNA genes obtained in this study were deposited in the DDBJ/EMBL/GenBank databases under accession numbers AB695349 through AB695357.

### Chemicals

4-Aminopyridine and methyl chloroformate were purchased from Tokyo Chemical Industry (Tokyo, Japan). 4-Amino-3-hydroxypyridine hydrochloride was from SynChem OHG (Felsberg, Germany). L-Mimosine from Koa Hoale seeds and pentafluorobenzyl bromide were from Sigma Aldrich (St. Louis, MO, USA). 3,4-Dihydroxypyridine was prepared from L-mimosine according to a previously reported method [23]. The ^1^H-NMR spectrum of the prepared 3,4-dihydroxypyridine was measured at NMR *δ*H (DMSO-*d*_6_): dH = 7.35 ppm (d, J = 6.0 Hz, 1H; H-6); 7.47 ppm (S, 1H; H-2); 6.21 ppm (d, J = 6.0 Hz; H-5). *N,O*-bis(trimethylsilyl)trifluoroacetamide and pyridine derivatives were purchased from Wako Pure Chemicals (Osaka, Japan).

## Results

### Degradation of 4-aminopyridine by the enrichment culture

**Figure 2:**
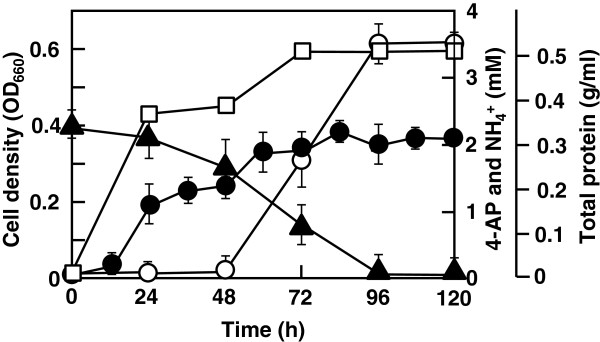
**Growth of the enrichment culture in medium containing 4-aminopyridine.** Growth and degradation of 4-aminopyridine. The enrichment culture was cultivated in medium containing 2.13 mM 4-aminopyridine (0.02% wt/vol) at 30°C with shaking. Growth was determined by measuring the optical density at 660 nm (OD_660_) (open squares); the residual 4-aminopyridine (filled triangles, 4-AP) was measured using HPLC as described in the text; the released ammonia (open circles) was measured using the indophenol method [21]; and total protein in the culture (filled circles) was measured using the modified Lowry method, independently performed twice.

We selected one 4-aminopyridine-degrading enrichment culture from the ten enrichment cultures of soil samples incubated continuously with subculturing for 6 months. The enrichment culture grew well and could be maintained on basal medium containing 4-aminopyridine in the presence of soil extract. The culture degraded 4-aminopyridine and used it as a carbon and nitrogen source (Figure 2).

### Identification and degradation of metabolites from 4-aminopyridine

Two metabolites in the enrichment culture in medium containing 4-aminopyridine were detected using GC and GC-MS. The trimethylsilylated metabolites, compounds I and II, had GC retention times of 20.9 and 24.4 min, respectively. Compound I was detected in the culture on the first day and accumulated during the cultivation. Compound II accumulated temporarily and was gradually degraded during cultivation. The mass spectrum of trimethylsilylated compound I showed a molecular ion at *m/z* 254 (M^+^, relative intensity 81.3%). Major fragment ions appeared at *m/z* 239 (M^+^-CH_3_, 90%) and 73 ([Si(CH_3_)_3_]^+^, 100%). The mass spectrum of trimethylsilylated compound II showed a molecular ion at *m/z* 255 (M^+^, relative intensity 25.7%). Major fragment ions appeared at *m/z* 240 (M^+^-CH_3_, 59.9%), 182 (M^+^-Si(CH_3_)_3_, 1.1%), 147 ([(CH_3_)_2_Si = O–Si(CH_3_)_3_]^+^, 2.1%), and 73 ([Si(CH_3_)_3_]^+^, 100%). The GC retention times and MS spectra of trimethylsilylated compounds I and II agreed with those of trimethylsilylated authentic 4-amino-3-hydroxypyridine and 3,4-dihydroxypyridine, respectively.

Pyridines are metabolized into an organic acid, such as acetate, formate, or dicarboxylic acids [4]. The culture supernatant of the enrichment culture was mixed with pentafluorobenzyl bromide and then analyzed. The mass spectrum of the pentafluorobenzyl derivative showed a molecular ion at *m/z* 226 (M^+^). The GC retention time and MS spectrum of the derivatized compound agreed with those of formate derivatized by pentafluorobenzyl bromide. In the enrichment cultures grown on 2.12, 6.38, and 10.6 mM 4-aminopyridine for 10 days, 0.05 ± 0.012 mM formate accumulated in 10.6 mM 4-aminopyridine medium.

Although the enrichment culture gradually degraded 4-aminopyridine with growth, 4-amino-3-hydroxypyridine accumulated in the culture to a final concentration of 6.4 × 10^−3^ mM after 5 days of cultivation. When we cultivated the enrichment culture in basal medium containing 4-amino-3-hydroxypyridine or 3,4-dihydroxypyridine (final concentration, 0.05% wt/vol) with and without 4-aminopyridine, the culture completely degraded 3,4-dihydroxypyridine in both media in 4 days but did not degrade 4-amino-3-hydroxypyridine in either medium.

### Identification of the gene encoding 3-hydroxy-4-pyridine dioxygenase in the isolated strains

We hypothesized that 4-aminopyridine is metabolized to 3,4-dihydroxypyridine, and that the pyridine ring is then cleaved by 3-hydroxy-4-pyridone dioxygenase, as described below. The fragment amplified by *pydA*-specific primers was isolated and analyzed to determine whether some predominant strain in the enrichment culture carries the dioxygenase gene. The same sequence fragment was amplified from three different samples. The amino acid sequence deduced from the determined 801-bp sequence showed a high level of identity with sequences of the extradiol-dioxygenase-3B-like superfamily of proteins, especially with that of the putative PydA from *Hyphomicrobium* sp. MC1 (YP_004673996) (see Additional file 2: Figure S1).

### Isolation of culturable bacterial strains from the enrichment culture

The enrichment culture contained at least seven strains of dominant bacteria (designated as strains 4AP-A to 4AP-G) that could grow on nutrient agar. The physiological and biochemical parameters of strains 4AP-A and 4AP-G were characterized, and their 16S rRNA genes were analyzed by sequencing. Strains 4AP-B, 4AP-C, 4AP-D, 4AP-E, and 4AP-F were classified by 16S rRNA gene analysis (Table 2, see Additional file 1: Tables S1 and S2). None of these strains could degrade 4-aminopyridine by itself or when combined with other strains, including all six of the other culturable dominant strains.

**Table 2:** Identification of bacteria constituting the 4-aminopyridine-degrading enrichment culture

**Strain**	**Genus or species affiliation (RDP II classifier)**	**Best database match**	**Identity (%)**
4AP-A	*Pseudomonas nitroreducens*	*P. nitroreducens* IAM 1439 (AM088473)	1511/1523 (99.1%)
4AP-B	*Stenotrophomonas maltophilia*	*S. maltophilia* e-p13 (AJ293473)	1532/1537 (99.7%)
4AP-C	*Enterobacter agglomerans*	*E. agglomerans* JCM1236 (AB004691)	1514/1536 (99.3%)
4AP-D	*Tsukamurella pulmonis*	*T. pulmonis* NIPHL170804 (AY741505)	1505/1515 (99.1%)
4AP-E	*Burkholderia*	*B. cenocepacia* J2315 (AM747721)	1523/1525 (99%)
4AP-F	*Microbacterium*	*M. esteraromaticum* S29 (AB099658)	1509/1519 (99%)
4AP-G	*Enterobacter*	*Enterobacter* sp. SPh (FJ405367)	1494/1501 (99%)
4AP-Y	*Hyphomicrobium*	Uncultured *Hyphomicrobium* sp. (FJ889298)	1427/1437 (99%)
4AP-Z	*Elizabethkingia*	*E. meningoseptica* R3-4A (HQ154560)	1043/1046 (99.7%)

When ten-fold-diluted enrichment culture was spread on agar plates containing 4-aminopyridine, several small colonies appeared. Colony PCR analysis of the 16S rRNA gene indicated that these were colonies of strains 4AP-A, identified as a species of *Pseudomonas* and 4AP-G, identified as a species of *Enterobacter*. Attempts to isolate 4-aminopyridine-degrading bacteria by changing the concentration of 4-aminopyridine and the incubation period at 30°C were unsuccessful. We could, however, isolate large colonies of strain 4AP-A on an agar plate containing 3,4-dihydroxypyridine.

### DGGE analysis of the enrichment culture

**Figure 3:**
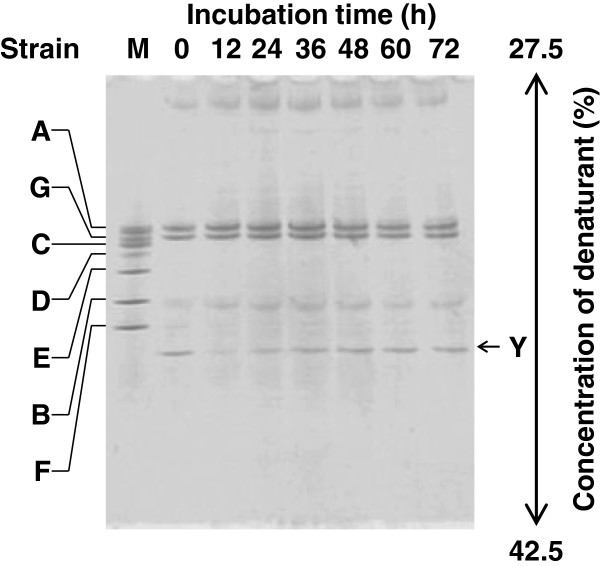
**DGGE profile of the enrichment culture during cultivation in medium containing 4-aminopyridine.** Standard amplified fragments from strains 4AP-A, 4AP-B, 4AP-C, 4AP-D, 4AP-E, 4AP-F, and 4AP-G were loaded in lane M. The enrichment culture grown in medium containing 4-aminopyridine was used to inoculate fresh medium (0.5 ml) containing 2.13 mM 4-aminopyridine (0.02% wt/vol), and the subculture was incubated at 30°C with shaking. The subculture was sampled (0.8 ml) every 12 h, and the harvested cells were used for PCR-DGGE.

The enrichment culture grown in 2.13 mM 4-aminopyridine medium was used to inoculate fresh medium containing 4-aminopyridine, and aliquots of the new, growing culture were collected in the early-, mid-, and late-exponential growth phases as described in the Materials and methods section. In DGGE gels, the intensity of the bands of some samples increased with the degradation of 4-aminopyridine, and two main bands were present at the same intensity in all samples throughout growth (Figure 3). These two main bands were assigned to strains 4AP-A and 4AP-G based on sequence analysis of the V3 regions of the 16S rRNA gene from those two main bands and of the complete 16S rRNA gene from culturable strains 4AP-A to 4AP-G.

**Figure 4:**
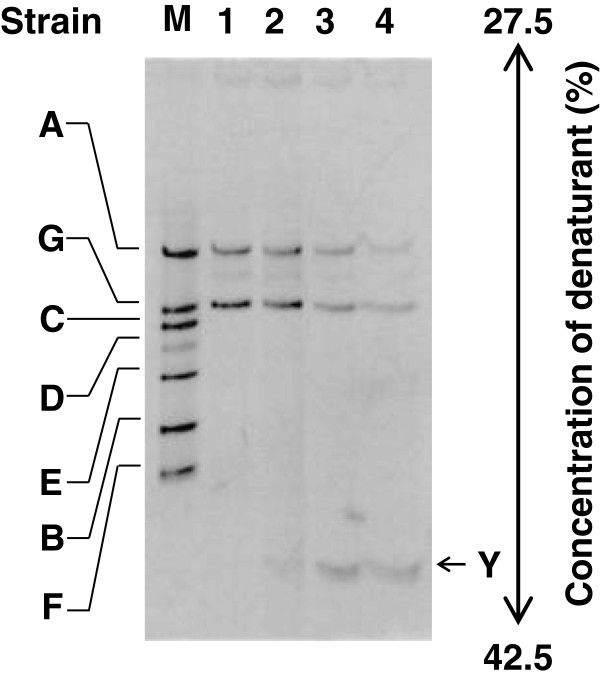
**DGGE profile of the enrichment culture grown in media containing various concentrations of 4-aminopyridine.** The enrichment culture was used to inoculate basal medium without 4-aminopyridine (lane 1) and with 4-aminopyridine (lane 2, 2.13 mM; lane 3, 10.6 mM; and lane 4, 53.2 mM), and the subcultures were incubated at 30°C with shaking. After 4 days of cultivation, the subcultures were sampled for PCR-DGGE analysis. The standard amplified fragments from strains 4AP-A, 4AP-B, 4AP-C, 4AP-D, 4AP-E, 4AP-F, and 4AP-G were loaded in lane M.

We then cultivated the enrichment culture in medium containing various concentrations of 4-aminopyridine to reveal the effect of the compound on the abundance of the dominant bacteria. The intensity of a new band (assigned to strain 4AP-Y) increased with the 4-aminopyridine concentration (Figure 4), whereas the intensity of the bands assigned to strains 4AP-A and 4AP-G decreased.

**Figure 5:**
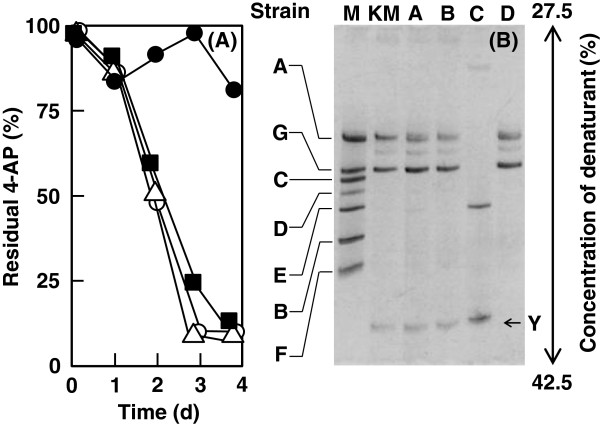
**DGGE profile of the enrichment cultures from a diluted pre-culture sample.** (**A**) Degradation of 4-aminopyridine by the diluted enrichment culture. The enrichment culture grown in medium containing 4-aminopyridine was diluted 10^8^-fold with 0.8% NaCl solution, and the diluted culture was used to inoculate fresh medium containing 2.13 mM 4-aminopyridine; the subculture was incubated at 30°C with shaking. The remaining 4-aminopyridine (4-AP) was measured using HPLC as described in the text. (Subcultures: a, open triangles; b, open circles; and c, filled squares; d, filled circles). The results of one representative experiment are shown; the residual 4-aminopyridine was measured in triplicate. (**B**) DGGE profiles of the enrichment culture. Subcultures that degraded 4-aminopyridine in 4 days (a, b, and c) and the subculture that did not degrade 4-aminopyridine (d) were analyzed by PCR-DGGE. The standard amplified fragments from strains 4AP-A, 4AP-B, 4AP-C, 4AP-D, 4AP-E, 4AP-F, and 4AP-G were loaded in lane M. The harvested cells of the enrichment culture were also used for PCR-DGGE (lane KM).

To clarify the role of strain 4AP-Y in the biodegradation of 4-aminopyridine, we diluted the enrichment culture 10^8^-fold in 0.8% (wt/vol) NaCl solution and used it to inoculate 40 tubes of medium containing 2.13 mM 4-aminopyridine, yeast extract, and soil extract. The optical density at 660 nm gradually and similarly increased in all subcultures. However, the rates of 4-aminopyridine degradation in the 40 subcultures differed. We compared the bacteria in the three subcultures that completely degraded 4-aminopyridine in 4 days (Figure 5A, subcultures a, b, and c) with the subculture that did not degrade the substrate (Figure 5A, subculture d). DGGE analysis showed that those subcultures that degraded 4-aminopyridine contained strain 4AP-Y as a predominant strain (Figure 5B, subcultures a, b, and c), whereas the subculture that did not degrade 4-aminopyridine did not contain strain 4AP-Y (Figure 5B, subculture d).

The full-length sequence of the 16S rRNA gene of strain 4AP-Y showed a high level of identity with that of a *Hyphomicrobium* species detected in a waste-treatment plant (AF098790, [24]) and of unculturable *Hyphomicrobium* species detected by PCR-DGGE (FJ889298, 4; FJ536932, [25]) (Additional file 1: Table S2). Species of the genus *Hyphomicrobium* form characteristic mother cells with hyphae and can utilize C1 compounds, e.g., methanol, formate, or methylamine [26]. We observed bi-polar filamentous cells with this shape in the culture grown with 4-aminopyridine (see Additional file 2: Figure S2). Our attempts to isolate *Hyphomicrobium* sp. strain 4AP-Y using medium containing methanol, formate, or formamide according to previously reported methods [26] failed.

**Figure 6:**
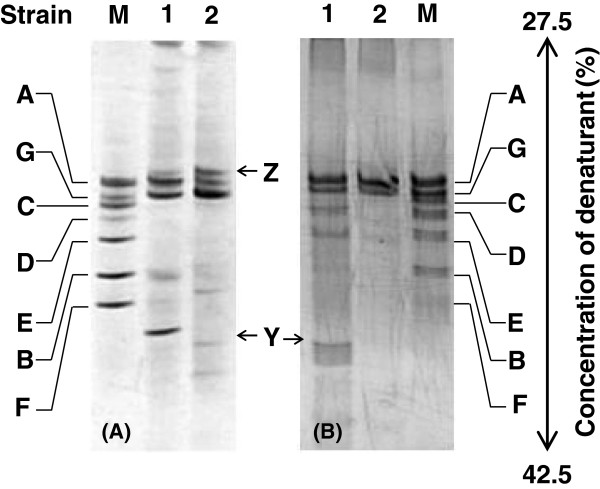
**DGGE profiles of the enrichment culture grown in medium containing 4-aminopyridine, 3,4-dihydroxypyridine, or formate.** The enrichment culture grown in medium containing 4-aminopyridine was used to inoculate medium containing 0.9 mM 3,4-dihydroxypyridine or 2.13 mM formate and 0.43 mM ammonium chloride. The culture was incubated and subcultured in fresh medium twice before DGGE analysis. (**A**) The standard amplified fragments from strains 4AP-A, 4AP-B, 4AP-C, 4AP-D, 4AP-E, 4AP-F, and 4AP-G were loaded in lane M. Lane 1, culture grown in medium containing 4-aminopyridine; lane 2, culture grown in medium containing 3,4-dihydroxypyridine. (**B**) The standard amplified fragments from the seven strains; lane 1, culture grown in medium containing formate and ammonium chloride; lane 2, culture grown in medium in the absence of formate. Extraction of genomic DNA and preparation of DGGE samples were carried out in triplicate. Prominent DNA bands from the DGGE gels were extracted and used as PCR templates as described in the text.

We analyzed the bacteria in a culture grown with 3,4-dihydroxypyridine by PCR-DGGE (Figure 6A). The culture completely degraded 3,4-dihydroxypyridine during 4 days of cultivation. Among the dominant bacteria, strain 4AP-A grew well in the 3,4-dihydroxypyridine medium and completely degraded 3,4-dihydroxypyridine during 3 days of cultivation. Strain 4AP-G grew slowly and degraded the substrate in 7 days. In the DGGE gels, several bands, including that of strain 4AP-A, were present; the band corresponding to strain 4AP-Y was absent; and a new band appeared. The sequence of the 16S rRNA gene of the bacterium corresponding to the new band, strain 4AP-Z, showed a high level of identity with those of *Elizabethkingia* spp. (GU084120 and AY468482). We also analyzed the bacteria in a culture grown with formate by PCR-DGGE (Figure 6B). In the DGGE gels, several bands, including that of strain Y, were present.

## Discussion

The pyridine-ring hydroxylation step is one of main initial steps in the degradation of pyridines [4]. Our analyses of the accumulated metabolites from 4-aminopyridine and the growth substrate specificity suggested that 4-aminopyridine was converted to 4-amino-3-hydroxypyridine and 3,4-dihydroxypyridine (Figure 1). We hypothesized that 4-hydroxypyridine is another possible metabolite based on the previously reported metabolic pathways of pyridines [3]. The enrichment culture could not degrade 4-amino-3-hydroxypyridine and 4-hydroxypyridine, even when 4-aminopyridine was added to the medium. Therefore, 4-amino-3-hydroxypyridine must be a dead-end product. In the enrichment culture, 4-aminopyridine probably would be directly converted to 3,4-dihydroxypyridine mainly by dehydroxylation and the release of ammonia (Figure 1), similar to the conversion of aniline to benzenediol (catechol) by a dioxygenase [27].

How 3,4-dihydroxypyridine is further metabolized in the enrichment culture is not known, but in *Agrobacterium* sp. NCIB 10413, 3,4-dihydroxypyridine is converted to 3-formiminopyruvate via the putative intermediate 3-(*N*-formyl)-formiminopyruvate by the *N*-heterocyclic ring-cleavage dioxygenase, 3-hydroxy-4-pyridone dioxygenase (3,4-dihydroxypyridine 2,3-dioxygenase) [6,7]. The gene encoding 3-hydroxy-4-pyridone dioxygenase, *pydA*, from *Rhizobium* sp. TAL1145 has been cloned, and the *pyd* gene cluster (AY729020) involved in the degradation and transport of 3-hydroxy-4-pyridone has been functionally analyzed [28]. However, the dioxygenases from strains NCIB 10413 and TAL1145 have not yet been purified and characterized. This enzyme is unstable and easily loses activity during cell extract preparation [6,7]. PydA from strain TAL1145 shows a high level of sequence identity with previously reported class III type *meta*-cleavage dioxygenases including putative 3-hydroxy-4-pyridone dioxygenase (YP_004673996) from *Hyphomicrobium* sp. MC1. Here, we did not detect dioxygenase activity in the mixed cells harvested from the enrichment culture. In a preliminary study, the partial *pydA* gene fragment could be amplified from the cells by using *pydA*-specific primers. In future studies, we plan on sequencing the entire gene and analyzing its expression with northern blots instead of detecting dioxygenase activity, to obtain support for our proposed metabolic pathway for 4-aminopyridine.

DGGE analyses indicated that *Hyphomicrobium* sp. strain 4AP-Y is a prominent degrader of 4-aminopyridine in the enrichment culture (Figures 3, 4, and 5) and that strain 4AP-Y is outnumbered in 3,4-dihydroxypyridine medium (Figure 6A). Therefore, strain 4AP-Y probably converts 4-aminopyridine to 3,4-dihydroxypyridine (Figure 1). 3,4-Dihydroxypyridine, which is also formed from L-mimosine by intestinal bacteria, can be degraded by a much wider range of soil bacteria and ruminal bacteria than has been recognized previously [23,29,30]. 3,4-Dihydroxypyridine might be more easily degraded than 4-aminopyridine by the other strains in our enrichment culture, including strains 4AP-A and 4AP-Z (Figure 1).

*Hyphomicrobium* spp. closely related to strain 4AP-Y have been isolated from waste-water plants [24] or detected as unculturable bacteria by PCR-DGGE [25,31]. Species of the genus *Hyphomicrobium* are oligocarbophilic and can grow on mineral salt medium, and the growth can be stimulated by soil extract [26]. In addition, they grow well on C1 compounds, such as methanol, methylated amines or formate [26]. However, little is known about the assimilation of aromatic compounds by *Hyphomicrobium* spp. [32]. The unculturable *Hyphomicrobium* sp. Y17-2 becomes numerically dominant in enrichment cultures containing toluene and *o*-xylene [33]. In our enrichment culture, *Hyphomicrobium* sp. 4AP-Y probably plays an important role in the initial step of 4-aminopyridine degradation. Other dominant strains, such as strains 4AP-A and 4AP-Z probably share 3,4-dihydroxypyridine and its metabolites as growth substrates. Strain 4AP-Y probably utilizes one of final metabolites from 3,4-dihydroxypyridine, i.e., formate, via the further degradation of this intermediate by other dominant strains.

The phytotoxicity, absorption, and translocation of 4-aminopyridine in corn and sorghum growing in treated nutrient cultures and soils have been examined by Starr and Cunningham [34]. Although aerobic and anaerobic degradation of 4-aminopyridine in soil had been expected, the authors found little evidence to support biodegradation. Our data reported here indicated that 4-aminopyridine can be mineralized by soil microbiota, and we identified bacteria possibly involved in the degradation. To further elucidate the degradation, we will need to establish culture conditions for the isolation of strain 4AP-Y to be able to study the enzymes involved in the degradation of 4-aminopyridine.

## Conclusions

We isolated a 4-aminopyridine-degrading enrichment culture from a normal soil sample, revealed the metabolic fate of 4-aminopyridine, and characterized the bacterial population in the culture. GC-MS analysis and growth substrate specificity indicated that 4-aminopyridine was probably metabolized to 3,4-dihydroxypyridine and that formate probably is one of metabolites. DGGE analysis revealed that the unculturable strain, *Hyphomicrobium* sp. strain 4AP-Y became more dominant with increasing 4-aminopyridine concentration in the culture and in the presence of formate and *Elizabethkingia* sp. 4AP-Z was dominant in the presence of 3,4-dihydroxypyridine. *Hyphomicrobium* sp. strain 4AP-Y, *Elizabethkingia* sp. 4AP-Z, and the culturable 3,4-dihydroxypyridine-degrading bacterium, *Pseudomonas nitroreducens* 4AP-A and *Enterobacter* sp. 4AP-G probably play important roles in 4-aminopyridine degradation.

## Competing interests

The authors declare that they have no competing interests..

## Authors’ contributions

All authors contributed in the organization and design of experiments as well as data interpretation and manuscript preparation. RN and AM isolated the 4-aminopyridine-degrading enrichment culture and identified the culturable bacteria. RN performed the DGGE analysis. ST separated and identified the metabolites. ST and KY wrote the manuscript. All authors read and approved the final version of the manuscript.

## Supplementary Material

Additional file 1: Table S1 Identification of strains in the 4-aminopyridine-degrading enrichment culture. **Table S2.** 16S rRNA gene analysis of the predominant bacteria in the 4-aminopyridine-degrading enrichment culture. Click here for file

Additional file 2: Figure S1 Alignment of the partial sequence of the putative 3-hydroxy-4-pyridone dioxygenase (PydA) from 3,4-dihydroxypyridine-degrading bacteria with sequences of previously reported PydAs. **Figure S2.** Micrograph of cells of the enrichment culture growing in medium containing 4-aminopyridine. Click here for file
