# Supplementary material for: Enrichment and characterization of a bacterial culture that can degrade 4-aminopyridine
Source: BMC Microbiol. 2013 Mar 21;13:62. doi: 10.1186/1471-2180-13-62 (PMC3637104; doi:10.1186/1471-2180-13-62)
Supplement: Additional file 1: Table S1 — Identification of strains in the 4-aminopyridine-degrading enrichment culture. Table S2. 16S rRNA gene analysis of the predominant bacteria in the 4-aminopyridine-degrading enrichment culture. [file 1471-2180-13-62-S1.pdf]

Additional file 1

Enrichment and characterization of a bacterial culture that can degrade 4-aminopyridine

Shinji Takenaka[§], Ryosuke Nomura, Ayumi Minegishi and Ken-ichi Yoshida

Institutional address:

Department of Applied Biological Chemistry, Graduate School of Agricultural Science,

Kobe University, 1-1 Rokkodai, Nada-ku, Kobe, 657-8501, Japan

Email addresses:

Shinji Takenaka (hakko3@kobe-u.ac.jp)

Ryosuke Nomura (noRyo888@gmail.com)

Ayumi Minegishi (Ayumi.Minegishi@inx.co.jp)

Ken-ichi Yoshida (kenyoshi@kobe-u.ac.jp)

[§]**Correspondence:**

Shinji Takenaka (hakko3@kobe-u.ac.jp)

Department of Applied Biological Chemistry, Graduate School of Agricultural Science,
Kobe University, 1-1 Rokkodai, Nada-ku, Kobe, 657-8501, Japan

SUPPLEMENTAL TABLE S1

Identification of strains in the 4-aminopyridine-degrading enrichment culture.

Test or characteristic	Strain	
	4AP-A [a]	4AP-G
Morphology	Rod	Rod
Size (μM)	1.0×1.5–2.0	0.7-1.1×2.1–2.5
Gram stain	Negative	Negative
Catalase	+	+
Oxidase	+	-
Motility	+	+
OF test<br>(Utilized sugar) [d]	Oxidative<br>(D-Glu, D-Fru, D-Man, D-Gal, Lac)	Oxidative<br>(D-Fru, D-Gal, Sac)
Gelatin hydrolysis	+	+
Production of fluorescent pigment	+	N.D.
16S rRNA analysis Registered strain (Accession no., identity in %)	*Pseudomonas nitroreducens* DSM 14399 (AM088474, 99.1%)<br>*Pseudomonas multiresinivorans* ATCC 700690T (X96787, 99.1%)<br>*Pseudomonas acephalitica* Ind01 (AM407893, 99.0%)	*Enterobacter* sp. SPh (FJ405367, 99.5%),<br>*Enterobacter* sp. TUT1014 (AB098582, 99.5%),<br>*Enterobacter* sp. SPj (FJ405369, 99.2%)
Accession no.	AB695349	AB695355
Classification	*Pseudomonas nitroreducens*	*Enterobacter* sp.

[a] Strain 4AP-A was identified by specific properties, lack of gelatin hydrolysis, aesuculin hydrolysis, and production of fluorescent pigments during the early growth stage, similar to *P. nitroreducens*.

Reference

Lang E, Griese B, Spröer C, Schumann P, Steffen M Verbarg S: Characterization of '*Pseudomonas azelaica*' DSM 9128, leading to emended descriptions of *Pseudomonas citronellolis* Seubert 1960 (Approved Lists 1980) and *Pseudomonas nitroreducens* Iizuka and Komagata 1964 (Approved Lists 1980), including *Pseudomonas multiresinivorans* as its later heterotypic synonym. *Int Syst Evol Microbiol* 2007, 57(4):878–882.

SUPPLEMENTAL TABLE S2

16S rRNA analysis of the predominant bacteria in the 4-aminopyridine-degrading enrichment culture

Strain	Accession no.	Strain (accession no., identity in %)
4AP-A	AB695349	*Pseudomonas nitroreducens* DSM 14399 (AM088474, 99.1%) *Pseudomonas multiresinivorans* ATCC 700690T (X96787, 99.1%) *Pseudomonas acephalitica* Ind01 (AM407893, 99.0%)
4AP-B	AB695350	*Stenotrophomonas maltophilia* IAM 12423 (AB294553, 99.7%), *S. maltophilia* NCB0306-284 (AB294557, 99.7%), *S. maltophilia* e-p13 (AJ293473, 99.7%)
4AP-C	AB695351	*Enterobacter agglomerans* JCM1236 (AB004691, 99.3%), *E. cloacae* drh3 (AF157695, 99.2%), *E. ludwigii* EN-119 (AJ853891, 98.8%)
4AP-D	AB695352	*Tsukamurella tyrosinosolvens* DSM 44234 (AY238514, 99.3%), *T. strandjordii* ATCCBAA-173 (AF283283, 99.2%), *T. pulmonis* NIPHL17084 (AY741505, 99.1%)
4AP-E	AB695353	*Burkholderia cepacia* RRE5 (AY946011, 99.9%), *B. cepacia* CNR22 (AB114607, 99.8%) *Burkholderia cenocepacia* pp9q (FJ870663, 99.8%)
4AP-F	AB695354	*Microbacterium* sp. JCM 9635 (AB563788, 99.8%), *Microbacterium esteraromaticum* S29 (AB099658, 99.3%), *Microbacterium arabinogalactanolyticum* DSM 8611 (Y17228, 99.7%)
4AP-G	AB695355	*Enterobacter* sp. SPh (FJ405367, 99.5%), *Enterobacter* sp. TUT1014 (AB098582, 99.5%), *Enterobacter* sp. SPj (FJ405369, 99.2%)
4AP-Y	AB695356	Uncultured *Hyphomicrobium* sp. clone Plot18-2C07 (FJ889298, 99.3%), *Hyphomicrobium* sp. M3 (AF098790, 98.8%), *Hyphomicrobium facile* (AB222020, 98.6%), uncultured *Hyphomicrobium* sp. clone ENR25 (FJ536932, 99.4%)
4AP-Z	AB695357	*Elizabethkingia meningoseptica* strain R3-4A (HQ154560, 99.7%), uncultured *Chryseobacterium* sp. clone PW9 (DQ355178, 99.5%), *Elizabethkingia* sp. F3 (GU084120, 99.4%)
