# Supplementary material for: Enrichment and characterization of a bacterial culture that can degrade 4-aminopyridine
Source: BMC Microbiol. 2013 Mar 21;13:62. doi: 10.1186/1471-2180-13-62 (PMC3637104; doi:10.1186/1471-2180-13-62)
Supplement: Additional file 2: Figure S1 — Alignment of the partial sequence of the putative 3-hydroxy-4-pyridone dioxygenase (PydA) from 3,4-dihydroxypyridine-degrading bacteria with sequences of previously reported PydAs. Figure S2. Micrograph of cells of the enrichment culture growing in medium containing 4-aminopyridine. [file 1471-2180-13-62-S2.pdf]

Additional file 2

Enrichment and characterization of a bacterial culture that can degrade 4-aminopyridine

Shinji Takenaka[§], Ryosuke Nomura, Ayumi Minegishi and Ken-ichi Yoshida

Institutional address:

Department of Applied Biological Chemistry, Graduate School of Agricultural Science, Kobe University, 1-1 Rokkodai, Nada-ku, Kobe, 657-8501, Japan

Email addresses:

Shinji Takenaka (hakko3@kobe-u.ac.jp)

Ryosuke Nomura (noRyo888@gmail.com)

Ayumi Minegishi (Ayumi.Minegishi@inx.co.jp)

Ken-ichi Yoshida (kenyoshi@kobe-u.ac.jp)

[§]**Correspondence:**

Shinji Takenaka (hakko3@kobe-u.ac.jp)

Department of Applied Biological Chemistry, Graduate School of Agricultural Science,
Kobe University, 1-1 Rokkodai, Nada-ku, Kobe, 657-8501, Japan

```
PydA_KM20-14E     ------------------------------------------------------------
PydA_MC1          -----MAKVVTALSMSHAPGVLGWPDAPSQEMKDSVAAAHAECARRLQEAKPDVIIAFLD
PydA_TAL1145      MRRKLMAEVVCALSMSHAPGVLGWADAPSEAVKKSIHEAHQECARRLEAARPDLIIAFLD
PydA_RB50         -----MAKIVAGIGMSHAPGALGWPDAPSASVRRRLLQAADRLGRSLDAARPDVIIAFLD
PydA_12822        -----MAKIVAGIGMSHAPGALGWQDAPSASVRRRLLQAADRLGRSLDAARPDVIIAFLD


PydA_KM20-14E     ---EXHFRNLMPTFAVGIAESHSGPADYMMAALKFDQKVVIDGQPELGEAMLRGLVSKGF
PydA_MC1          DHFENHFRNLMPTFAVGIAQTHSGPADYMMEALKFDKKAVIDGQPELGEAMLRGLVGLGF
PydA_TAL1145      DHFENHFRNMMPGFAVGISASHSGPADYMMEALKFDEKVVIESNVEHAETLLRGLIKRNF
PydA_RB50         DHFENHFRSLMPTVGIGVADSHPGPATQWLEALRLTRQERFGGAPEIAERLLRSLVADGY
PydA_12822        DHFENHFRSLMPTVGIGVADSHPGPATQWLEALRLTRQERFGGAPEIAERLLRSLVADGY

PydA_KM20-14E     DVARWGEIEYGNNLLVPLKFLTPAFDIPVLPLYINVFSPPLPSMKRAYDFGKAVREIVA-
PydA_MC1          DVARWGEIEYGNNLLVPLKFLTPNFDVPVLPLYINVFSPPLPTMNRAYDFGVAVRKIVD-
PydA_TAL1145      DAARWGEIEYGNTLLVPWKLIRPQYDIPVIPIYTNVFSPPLPSMARAYDMGAAVRDIVER
PydA_RB50         DVARWGEIEYGNNLMVPWKLMAPRSAPAIIPVFTNVFSPPVMPYRRAYAFGAALRNAAE-
PydA_12822        DVARWGEIEYGNNLMVPWKLMAPRSAPAIIPVFTNVFSPPVMPYRRAYAFGAALRNAAE-

PydA_KM20-14E     RLPDKLRVAFLATGGLSHWPPVWTEGAPDDDEFLQRMKKYQTEGRHVAEEDPELYSDLAR
PydA_MC1          GLPSDLRVAFLATGGLSHWPPVWTEGAPEDDEFLMRMKRYQTEGRQVALEDPSLYSDLAS
PydA_TAL1145      ELPTGLRVAFLATGGLSHWPPVWTEGAPEDDQFLQRMKRYQSEGKHVAEEDPTLYSDLAA
PydA_RB50         ALDADLRVAFMATGGMSHWPPFWNDSSPEADAFLARMKAFQTHGKSVLEKDPHLLRDLAA
PydA_12822        ALDADLRVAFMATGGMSHWPPFWNDSSPEADAFLARMKAFQTHGKSVLEKDPHLLRDLAA

PydA_KM20-14E     YEIEMASKMQWPLRIQHPLVNAHWDREIIEAFGRGDVEALCAMTYEEVEEGGGHGGHEIL
PydA_MC1          YEIEMASKMQWPLRIQHPLVNAHWDREIIEAFAKGDAEKLRGMTYEEVEEGGGHGGHEIL
PydA_TAL1145      YEIEMADKMQWPLRHQHPLVNAAWDREIIEAFGRGDTEYLRNCTFESVEEGGGHGGHEIL
PydA_RB50         YEIEMAERNQWPLNSPHPLVNEAWDRQMLDALARGDVEFLCGLQYEDVKRDGGHGGQEII
PydA_12822        YEIEMAERNQWPLNSPHPLVNEAWDRQMLDALARGDVEFLCGLQYEDVKRDGGHRGQEII

PydA_KM20-14E     NWVALMGAMDGAAAKIIAYEPVTEWXCGMNH-------------------
PydA_MC1          NWVAVMGAMGGAPAHIVGYEPVMEWICGMGYIAYDPPLAQAA--------
PydA_TAL1145      NWMAVMGAMGGSKADVIGYEPVVEWICGMGYIAYDAAA------------
PydA_RB50         NWIELMGAMKGAPATLLEYEAVTEWICGMAYMDYGVAQAWPVNPLIKEQE
PydA_12822        NWIELMGAMKGAPATLLEYEAVTEWICGMAYMDYGVAQAWPVNPLIKEQE
```

Figure S1 Alignment of the partial sequence of the putative 3-hydroxy-4-pyridone dioxygenase (PydA) from 3,4-dihydroxypyridine-degrading bacteria with sequences of previously reported PydAs. Conserved amino acid residues are indicated by outlined letters. Abbreviations: PydA_KM20-14E, putative PydA isolated from our 4-aminopyridine-degrading enrichment culture (this study); PydA_MC1, putative PydA of *Hyphomicrobium* sp. MC1 (YP_004673996); PydA_TAL1145, PydA of *Rhizobium* sp. TAL1145 (AY729020); PydA_RB50, putative PydA of *Bordetella bronchiseptica* RB50 (NP_890665); and PydA_12822, putative PydA of *Bordetella parapertussis* 12822 (NP_885852).

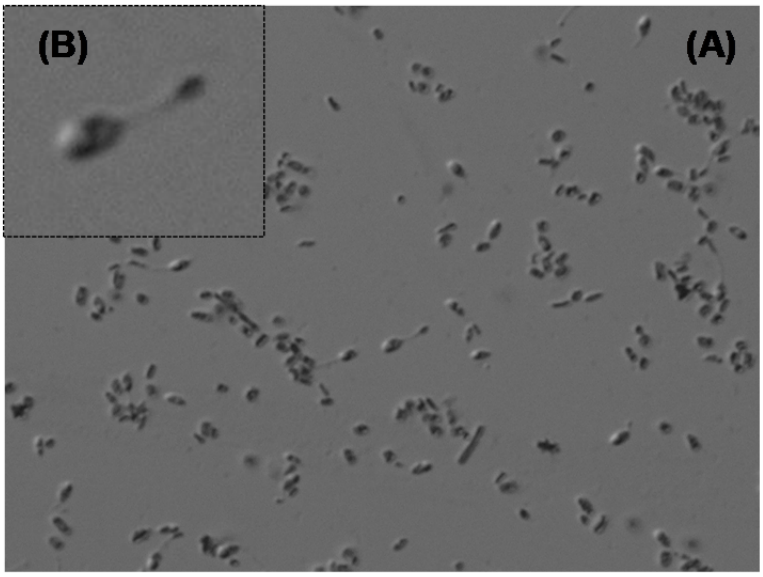

Figure S2 Micrograph of cells of the enrichment culture growing in medium containing 4-aminopyridine. (**A**) 4-Aminopyridine-degrading enrichment culture. (**B**) Enlargement of a cell shown in (A) showing the bipolar prosthecae morphology of the genus *Hyphomicrobium* .
